# P-261. Physician Attitude on Stethoscope Sanitization: A Quality Improvement Project

**DOI:** 10.1093/ofid/ofae631.465

**Published:** 2025-01-29

**Authors:** Lin Chang, Cilian J White

**Affiliations:** Morristown Medical Center - Atlantic Health System, Morristown, New Jersey; Morristown Medical Center, Morristown, New Jersey

## Abstract

**Background:**

Literature has demonstrated that stethoscopes can harbor pathogenic bacteria (*Staphylococcus aureus*, *Pseudomonas aeruginosa*, vancomycin-resistant *Enterococci* and *Clostridium difficile*) and act as vectors of infection transmission between patients. Although it seems most healthcare professionals are aware of the potential of stethoscopes to transmit infections, the practice of stethoscope cleaning varies widely and is often not up to standard recommendations. This QI project aims to determine current stethoscope sanitization practices at our hospital, Morristown Medical Center (MMC), barriers that prevent more frequent cleaning, and whether education and easier access to sanitization methods changes practices of providers.

**Figure 1:**
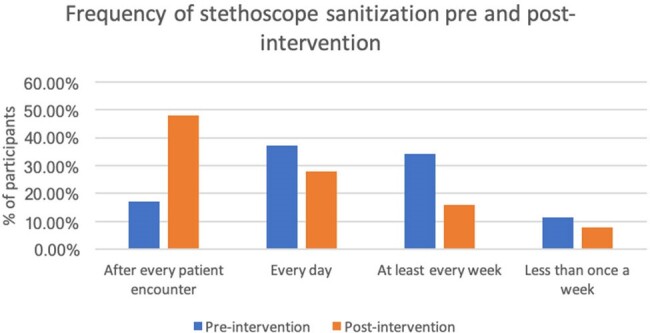
Frequency of stethoscope sanitization pre- and post-intervention

**Methods:**

Participants (physicians at MMC) were asked to complete a pre- and post-intervention survey about their stethoscope cleaning practices and barriers to more frequent cleaning. Intervention consisted of physician education and placement of alcohol wipes at physician workspaces for 3 weeks.

**Figure 2:**
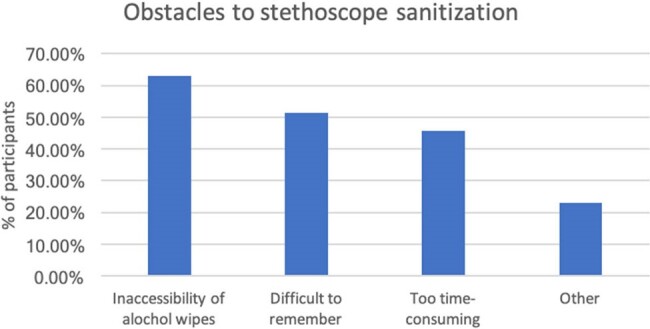
Obstacles to stethoscope sanitization

**Results:**

Of the 35 responses from the pre-intervention survey, 97% believed stethoscopes harbor harmful bacteria. 17% of physicians sanitized their stethoscopes after every patient encounter, 37% every day, 34% every week and 11.5% less than every week (Figure 1). Barriers to more frequent stethoscope cleaning included: inaccessibility of alcohol wipes (62.9%), difficult to remember (51.4%), too time-consuming (45.7%), and other (22.9%) (Figure 2). Post-intervention survey (25 responses) showed an increase from 17% to 48% for physicians that sanitize after every patient encounter. More importantly, 96% of participants agreed they would implement better stethoscope sanitization practices going forward.

**Conclusion:**

Physicians are aware of the potential for stethoscopes to harbor bacteria and transmit infections, however, their stethoscope cleaning practices do not necessarily align with this knowledge, with only 17% sanitizing after every patient encounter. Allowing easier access to alcohol wipes as well as physician education served to increase frequency of stethoscope sanitization overall. Our conclusion is that ongoing education and reminders can help improve overall stethoscope sanitization practices in the hospital.

**Disclosures:**

**All Authors**: No reported disclosures

